# Supplementary material for: RARRES3 suppresses breast cancer lung metastasis by regulating adhesion and differentiation
Source: EMBO Mol Med. 2014 May 27;6(7):865–81. doi: 10.15252/emmm.201303675 (PMC4119352; doi:10.15252/emmm.201303675)
Supplement: Supplementary file 1 — Supplementary Figure S1 [file emmm0006-0865-SD1.pdf]

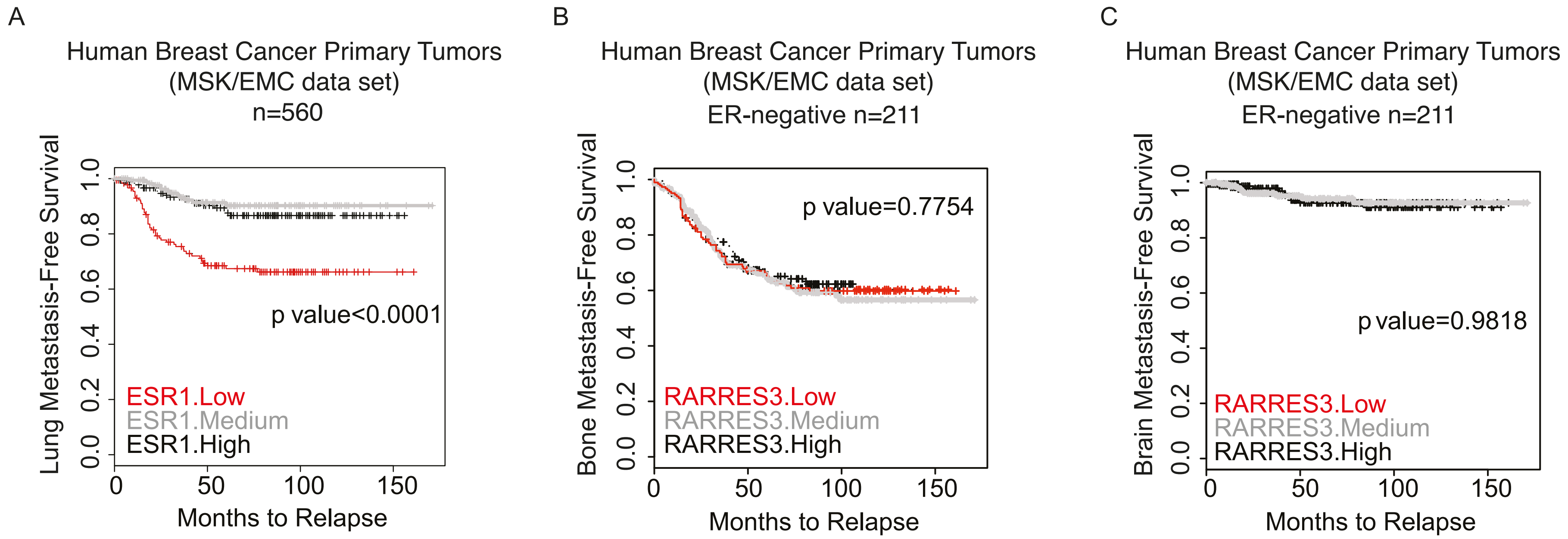

Supplementary Figure S1

Supplementary Figure S1

(A) Kaplan-Meier representation of the probability of lung metastasis-free survival in the MSK/EMC breast cancer tumor data set (n=560) according to *ESR1* levels of expression. Low, Med and High represent *ESR1* expression levels in the following way: low (<mean - SD), medium (≥ mean - SD and ≤ mean + SD) and high (> mean + SD).

(B) Kaplan-Meier representation of the probability of bone metastasis-free survival in the MSK/EMC breast cancer tumor data set (focused on ER-negative tumors n=211) according to *RARRES3* levels of expression. Low, Med and High represent *RARRES3* expression levels in the following way: low (<mean - SD), medium (≥ mean - SD and ≤ mean + SD) and high (> mean + SD).

(C) Kaplan-Meier representation of the probability of brain metastasis-free survival in the MSK/EMC breast cancer tumor data set (focused on ER-negative tumors n=211) according to *RARRES3* levels of expression. Low, Med and High represent *RARRES3* expression levels in the following way: low (<mean - SD), medium (≥ mean - SD and ≤ mean + SD) and high (> mean + SD).
