# Supplementary material for: RARRES3 suppresses breast cancer lung metastasis by regulating adhesion and differentiation
Source: EMBO Mol Med. 2014 May 27;6(7):865–81. doi: 10.15252/emmm.201303675 (PMC4119352; doi:10.15252/emmm.201303675)
Supplement: Supplementary file 2 — Supplementary Figure S2 [file emmm0006-0865-SD2.pdf]

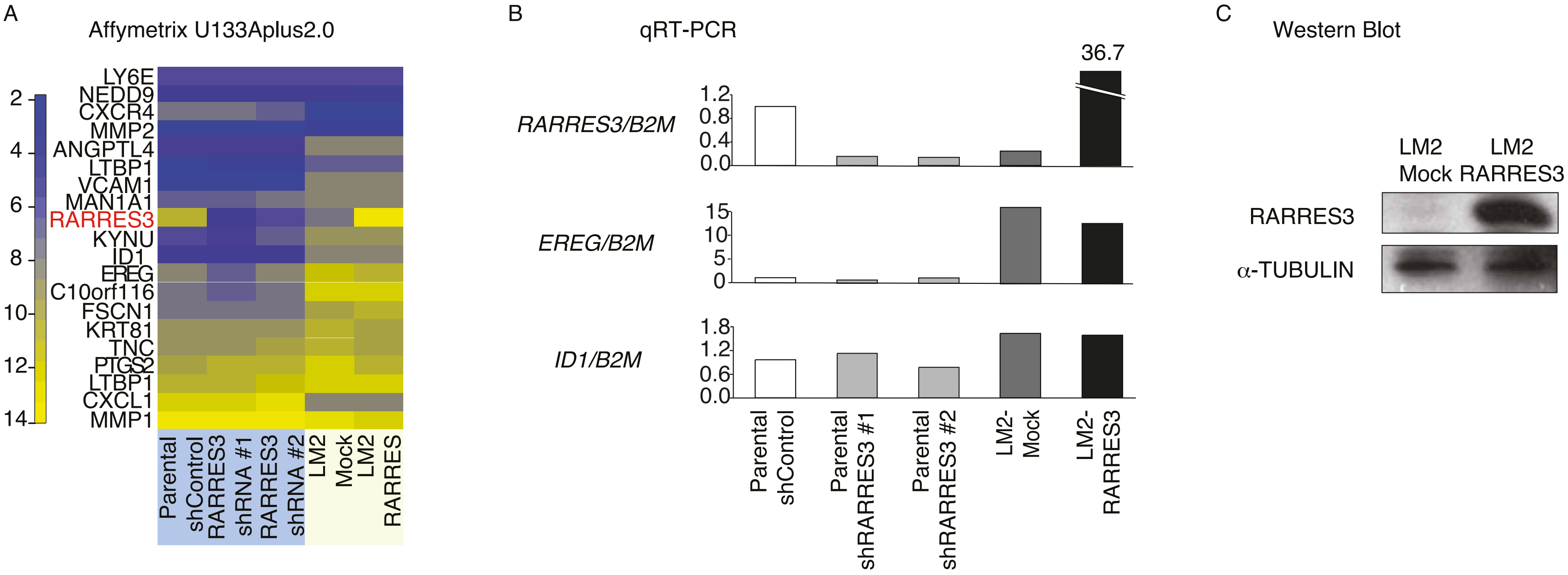

Supporting Information Figure 2

Supplementary Figure S2

(A) Knockdown of RARRES3 in Parental MDA-MB-231 cells and RARRES3 overexpression in LM2 metastatic derivatives as confirmed by gene expression profiling (Affymetrix U133APlus2.0). None of the other previously defined Lung Metastasis Signature Genes is consistently affected under the previous conditions.

(B) *RARRES3, EREG* and *ID1* mRNA expression levels measured by qRT-PCR and normalized to *B2M* levels. Data are presented as mean of three independent experiments.

(C) Western Blot analysis of RARRES3 levels in the indicated cell populations. α-TUBULIN was used as a loading control.
