# Supplementary material for: RARRES3 suppresses breast cancer lung metastasis by regulating adhesion and differentiation
Source: EMBO Mol Med. 2014 May 27;6(7):865–81. doi: 10.15252/emmm.201303675 (PMC4119352; doi:10.15252/emmm.201303675)
Supplement: Supplementary file 3 — Supplementary Figure S3 [file emmm0006-0865-SD3.pdf]

A

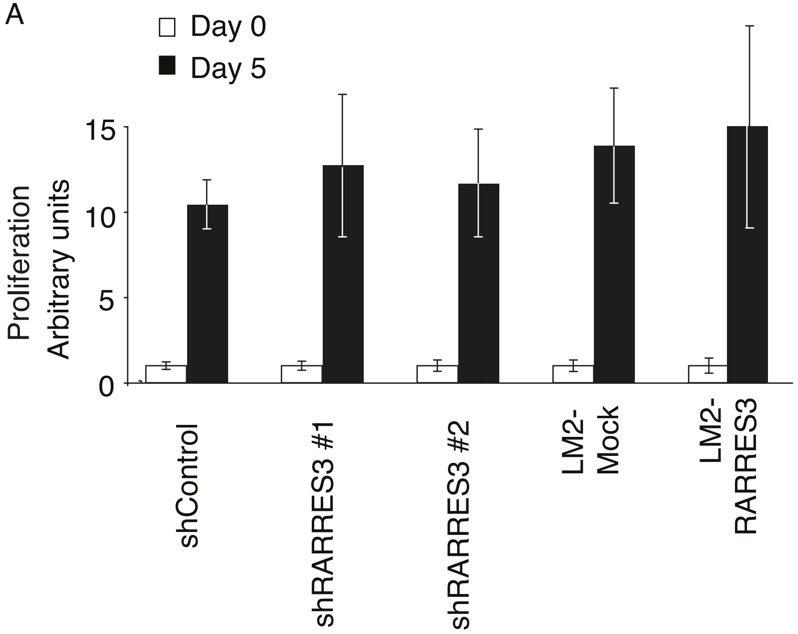

B

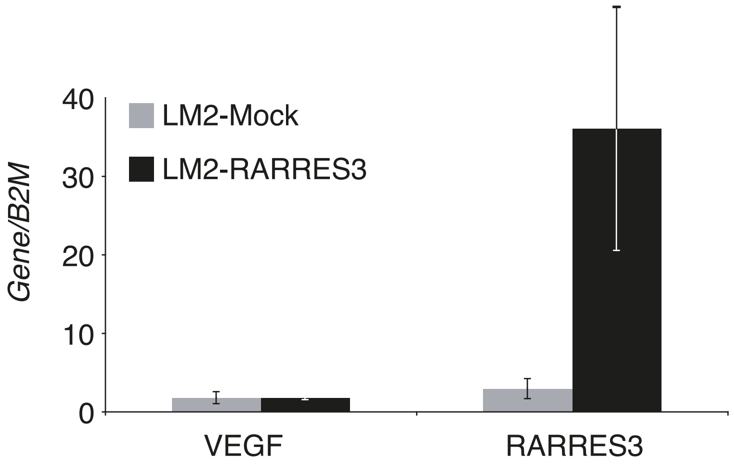

C

Dextran difusion in size-matched LM2 orthotopic MFP tumors

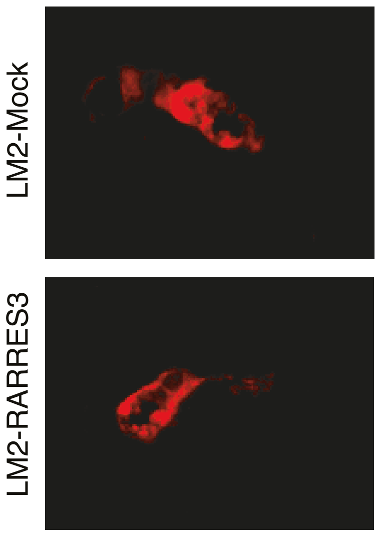

D

Peripheral Blood of mice bearing size-matched LM2 orthotopic MFP tumors

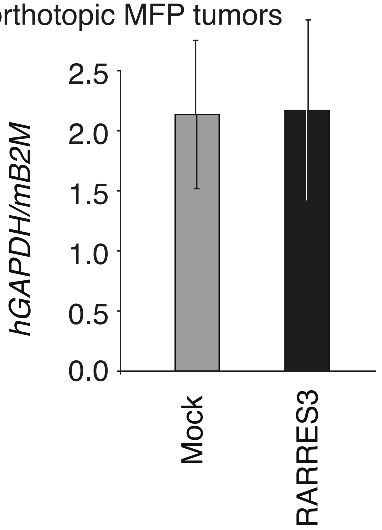

Supporting Information Figure 3

Supplementary Figure S3

(A) Proliferation assay. $5x10^4$ cells were seeded on day 0 and grown in regular media. At 5 days post-plating, cells were counted and normalized to day 0. Data are presented as mean of three independent experiments with SD.

(B) *VEGF* and *RARRES3* mRNA expression levels measured by qRT-PCR normalized to *B2M* levels. Data is presented as mean of three independent experiments with SD.

(C) Rhodamine conjugated dextran (70 KDa) was injected into mice bearing size-matched LM2 and LM2-RARRES3 mammary tumors. At 3 hours post-injection, mice were perfused to remove dextran from the vasculature, tumors were extracted, and sections were microscopically analyzed to detect dextran extravasation (five sections and n=5 per group). Representative images of LM2 and LM2-RARRES3 tumors are shown.

(D) Circulating human metastatic cells were measured by qPCR using a human B2M and mouse GAPDH mRNA probe in blood samples obtained from mice bearing mammary fat pad size-matched tumors of the indicated origin (n=5 per group). Data are averages ± SD.
