# Supplementary material for: RARRES3 suppresses breast cancer lung metastasis by regulating adhesion and differentiation
Source: EMBO Mol Med. 2014 May 27;6(7):865–81. doi: 10.15252/emmm.201303675 (PMC4119352; doi:10.15252/emmm.201303675)
Supplement: Supplementary file 4 — Supplementary Figure S4 [file emmm0006-0865-SD4.pdf]

A

		Parental			LM2	
		shControl	sh RARRES3#1	shRARRES3 #2	Mock	RARRES3
IHC	Ki-67 (% positive)					
	1ary Tumor	33,3 (±7.0)	39.3 (±8.5)	34.0 (±1.5)	24.5 (±0.6)	27.7 (±2.3)
	Lung Mets (7 weeks PI)	24.7 (±3.1)	28.5 (±5.3)	24.1 (±5.5)	28.4 (±1.7)	25.2 (±1.6)
	Caspase 3 (positive/field)					
	1ary Tumor	19.6 (±2.3)	25.6 (±2.3)	20.6 (±2.5)	N/A	N/A
in vivo	Caspase 3 (Arbitrary units)					
	Lung Mets (7 weeks PI)	17.4 (±5.4)	21.6 (±2.2)	27.1 (±4.7)	8.9 (±2.0)	9.3 (±1.9)

B

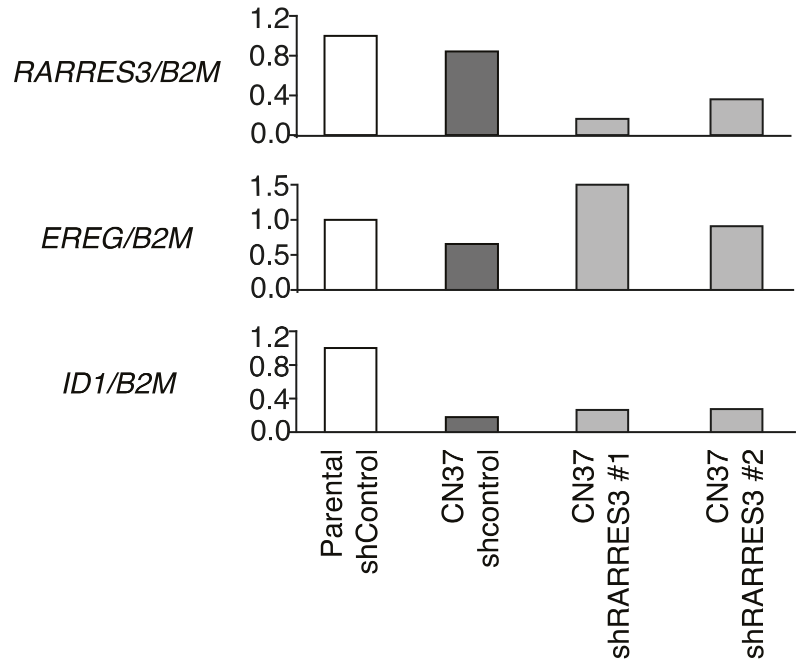

C

D

E

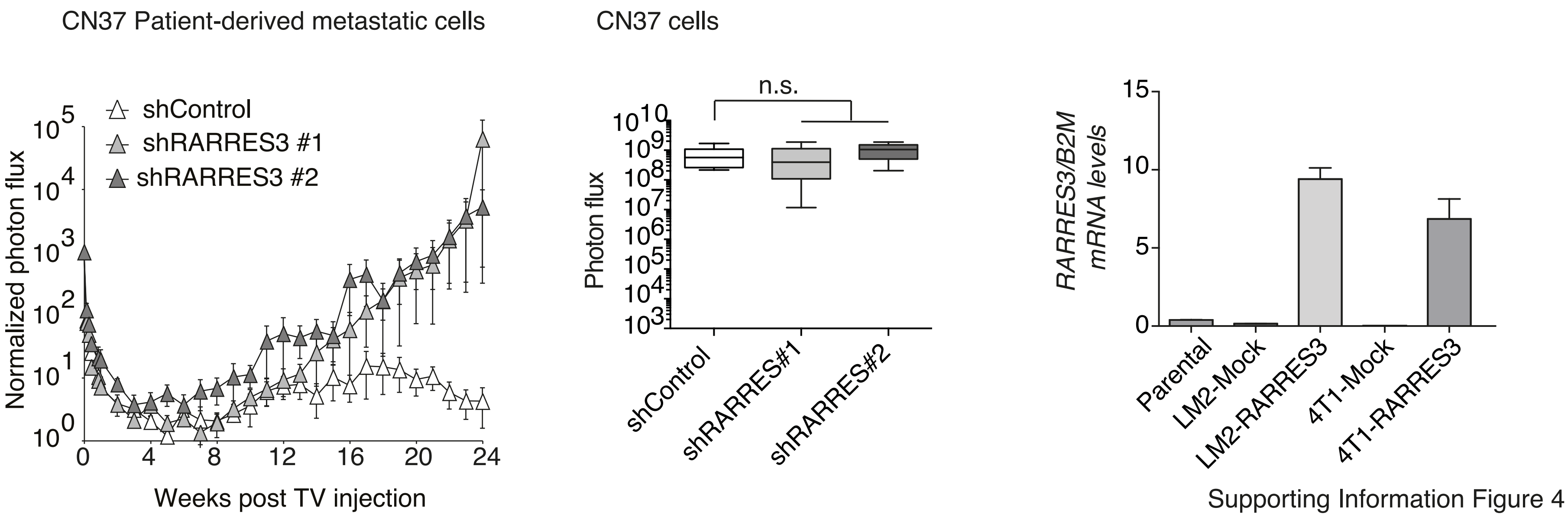

Supporting Information Figure 4

Supplementary Figure S4

(A) Percentage of Ki-67 positive cells in MFP tumors and lung metastasis 7 weeks post-inoculation were scored. Similarly, cleaved caspase-3 activity (arbitrary units) was determined by IHC in MFP tumors and lung metastasis 7 weeks post-inoculation. Five fields per section and 5 sections per lung/tumor were scored. Data are averages ± SD.

(B) *RARRES3, EREG* and *ID1* mRNA expression measured by qRT-PCR and normalized to *B2M* levels.

(C) CN37 cells ($2x10^5$) expressing a control vector (parental) or two independent RARRES3 shRNA vectors (shRARRES3 #1 and #2) were injected into the tail vein of mice. Lung colonization was assayed by weekly bioluminescence imaging. Plots show normalized photon flux in the lung over time (n=9, 9 or 8 per group, respectively).

(D) CN37 cells ($5x10^5$) expressing a shControl vector or two independent shRARRES3 vectors were implanted contra-laterally into the fourth mammary fat pad of mice, and tumor growth was measured at week 10 (end point) by photon flux determination. Data are averages ± SD (n=8 tumors per condition).

(E) *RARRES3* mRNA expression measured by qRT-PCR and normalized to *B2M* levels in Parental MDA-231, 4T1-Mock and 4T1-RARRES3 cells. Data are presented as mean of three independent experiments ± SD.
