# Supplementary material for: RARRES3 suppresses breast cancer lung metastasis by regulating adhesion and differentiation
Source: EMBO Mol Med. 2014 May 27;6(7):865–81. doi: 10.15252/emmm.201303675 (PMC4119352; doi:10.15252/emmm.201303675)
Supplement: Supplementary file 5 — Supplementary Figure S5 [file emmm0006-0865-SD5.pdf]

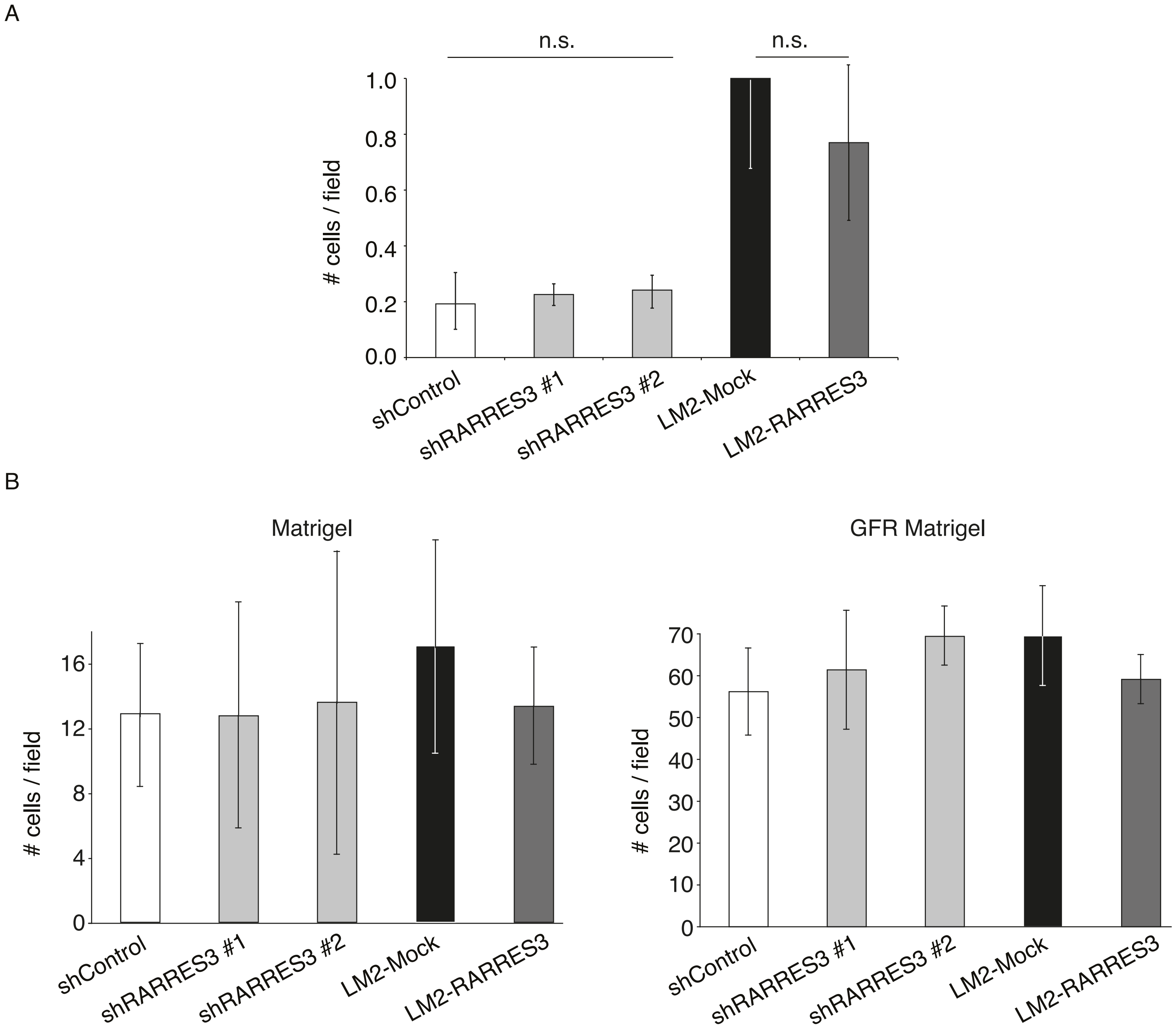

Supporting Information Figure 5

Supplementary Figure S5

(A) Migration of parental and LM2 cells expressing different levels of RARRES3 were scored in Boyden chambers covered with fibronectin. Each cell line was seeded in triplicate and 5 fields per chamber were counted. Results represent the average of three independent experiments ± SD

(B) Invasion of parental and LM2 cells expressing different levels of RARRES3 were scored in Boyden chambers covered with Matrigel (left panel) or growth factor-reduced Matrigel (right panel). Each cell line was seeded in triplicate, and 5 fields per chamber were counted. Results represent the average of three independent experiments ± SD
