# Supplementary material for: RARRES3 suppresses breast cancer lung metastasis by regulating adhesion and differentiation
Source: EMBO Mol Med. 2014 May 27;6(7):865–81. doi: 10.15252/emmm.201303675 (PMC4119352; doi:10.15252/emmm.201303675)
Supplement: Supplementary file 6 — Supplementary Figure S6 [file emmm0006-0865-SD6.pdf]

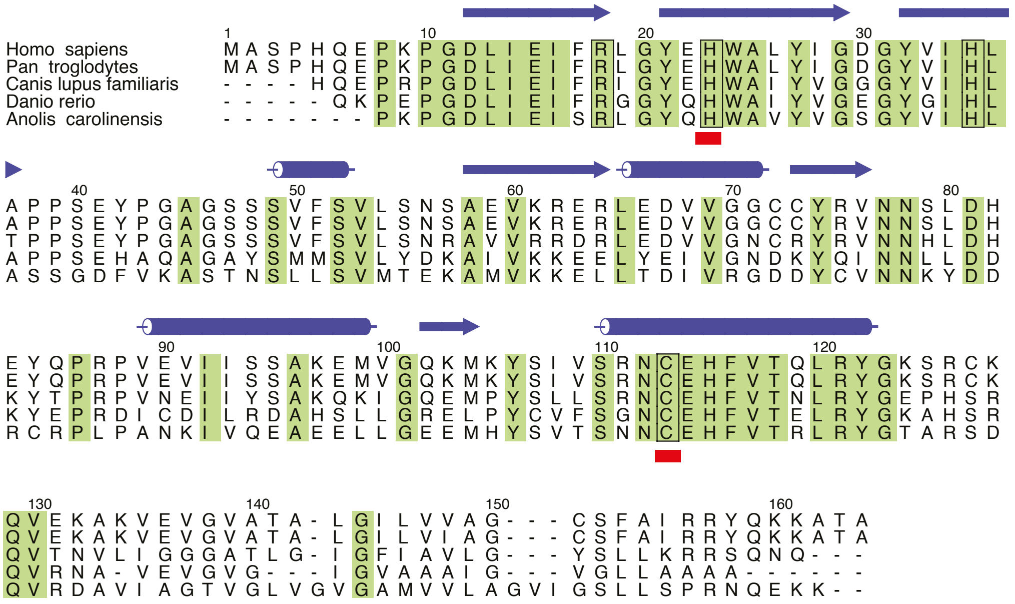

Mutant
RARRES-DEAD

Cys113Ser
His23Phe

Supporting Information Figure 6

Supplementary Figure S6

Sequence alignment of various RARRES3 homologous sequences showing conserved residues (green) and proposed active site residues (black boxes). The secondary structures of H-REV107 are shown above the alignment (purple).
