# Supplementary material for: RARRES3 suppresses breast cancer lung metastasis by regulating adhesion and differentiation
Source: EMBO Mol Med. 2014 May 27;6(7):865–81. doi: 10.15252/emmm.201303675 (PMC4119352; doi:10.15252/emmm.201303675)
Supplement: Supplementary file 7 — Supplementary Figure S7 [file emmm0006-0865-SD7.pdf]

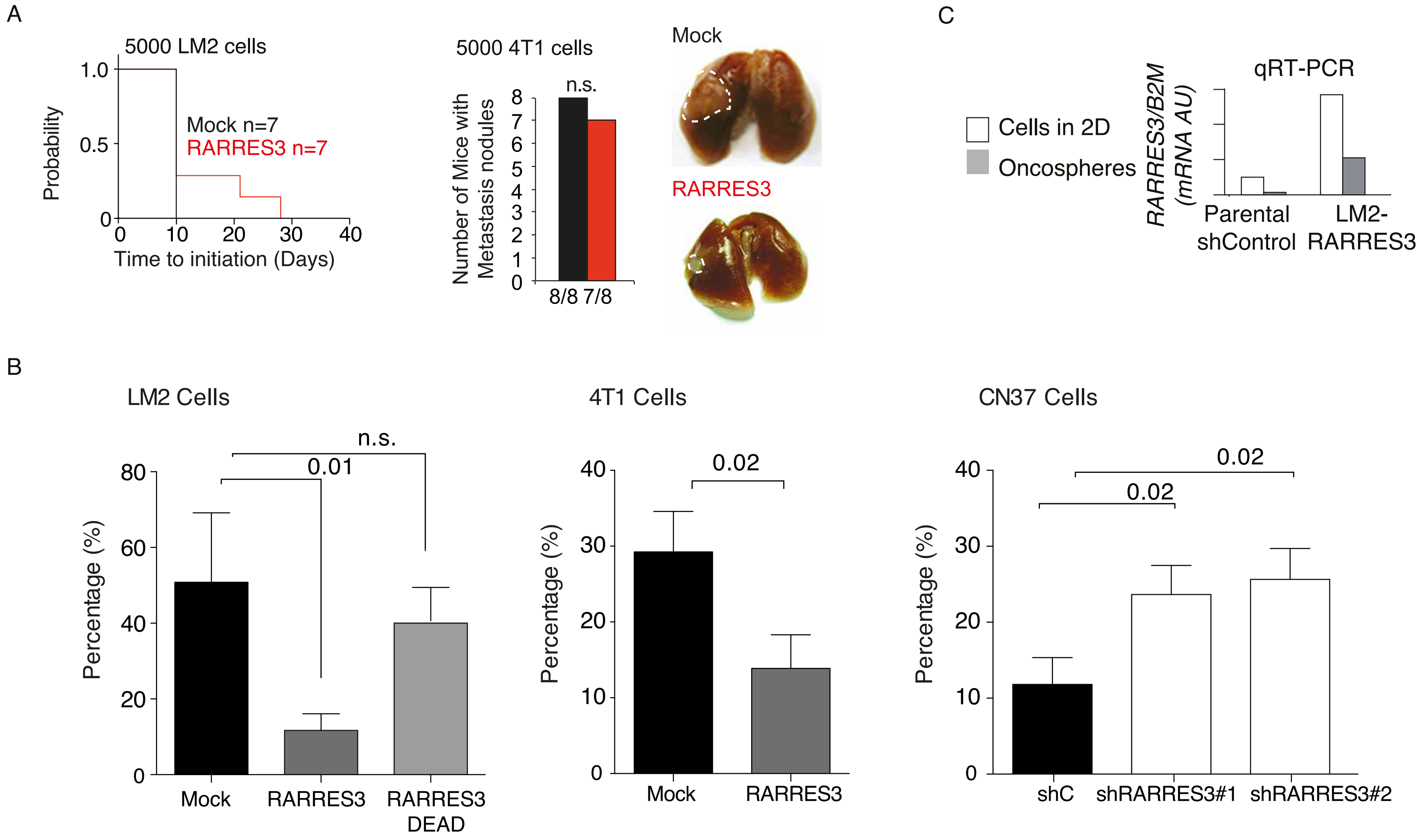

Supporting Information Figure 7

Supplementary Figure S7

(A) Mock and RARRES3-expressing LM2 and 4T1 cells were injected directly into mice lungs, and ability to grow in the lungs was assessed by luciferase bioluminescence over time or macroscopically 20 days post inoculation. Kaplan-Meier plot of the probability of lung metastasis-free survival was used for LM2 cells (log rank test) and a contingency plot was used for 4T1 cells (Fisher exact test) and representative images shown (white dashed line limits tumor area). n=7 mice per group was used in LM2 cells experiment. n=8 mice per group was used in 4T1 cells experiment.

(B) Equal dilutions of the indicated cell lines were performed, and cells were plated in Matrigel. The % of 3D oncospheres was calculated. The average of 3 experiments ± SD is shown.

(C) *RARRES3* mRNA qPCR analysis of the indicated cell lines either in attached culture (white bars) or in oncospheres culture (grey bars) is shown.
