# Supplementary material for: RARRES3 suppresses breast cancer lung metastasis by regulating adhesion and differentiation
Source: EMBO Mol Med. 2014 May 27;6(7):865–81. doi: 10.15252/emmm.201303675 (PMC4119352; doi:10.15252/emmm.201303675)
Supplement: Supplementary file 8 — Supplementary Table S1 [file emmm0006-0865-SD8.pdf]

Supplementary Table S 1. Clinical validation of potential metastasis suppressors described in the literature (Horack et al. 2008). In bold, those whose expression in the primary tumor predicts risk of site-specific relapse.

Gene symbol	Hazard Ratio of overall Mets	Hazard Ratio of Lung Mets*	Hazard Ratio of Brain Mets*	Hazard Ratio of Bone Mets
PEBP1	-1,10	**-1,44****	**-1,43**	-1,01
RARRES3	-1,03	**-1,31**	-1,01	1,02
SMAD7	-1,08	-1,20	-1,37	-1,05
ARHGDIB	-1,02	-1,16	1,21	1,04
BRMS1	-1,06	-1,15	-1,12	-1,03
CASP8	1,02	-1,15	1,02	1,01
CDH1	-1,06	-1,12	1,07	-1,03
MAP2K7	-1,06	-1,12	-1,24	-1,04
AKAP12	1,01	-1,09	1,11	1,04
CLDN4	-1,03	-1,09	1,07	1,04
SOCS3	-1,03	-1,06	-1,16	1,05
GPR68	1,01	-1,03	-1,19	1,06
MAP2K6	1,01	-1,02	-1,21	1,04
MAP2K4	-1,03	-1,01	1,18	-1,04
MATN2	-1.01	1.01	1.29	-1.11
MED23	1.04	1.02	-1.11	1.08
GSN	-1.03	1.06	1.16	-1.00
RECK	1.05	1.07	-1.06	1.01
CTGF	1.04	1.11	1.03	1.05
CD44	1.01	1.11	1.13	-1.01
DCC	1.06	1.13	1.25	1.07
NME1	-1.00	1.16	1.24*	-1.13
KISS1	1.08	1.18	**1.67**	1.06
DLC1	1.07	1.21*	-1.04	1.11
DRG1	1.04	**1.33***	1.01	-1.10
CD82	1.04	**1.39**	1.26	-1.03

*p<0.01
**p=0.019
