# Supplementary material for: RARRES3 suppresses breast cancer lung metastasis by regulating adhesion and differentiation
Source: EMBO Mol Med. 2014 May 27;6(7):865–81. doi: 10.15252/emmm.201303675 (PMC4119352; doi:10.15252/emmm.201303675)
Supplement: Supplementary file 9 — Supplementary Materials and Methods [file emmm0006-0865-SD9.pdf]

Supplementary Materials and Methods

Description of the MSK/EMC data set

We used the EMC-344 and MSK-82 data sets, which are based on HG-U133A and were combined, and also the EMC-189 data set, which is based on HG-U133plus2 and was processed separately (GSE2603, GSE12276, GSE5327, and GSE2034 available at the Gene Expression Omnibus (GEO) public database). In order to remove systematic biases, prior to merging the sets, the expression measurements were converted to z-scores for all genes. Patient clinical records of the 615 primary tumor samples has been extracted from the supplemental material described in Zhang, X.H., et al "Latent bone metastasis in breast cancer tied to Src-dependent survival signals" Cancer Cell. 2009, 6: 67-78. Following the indications of the Cancer Cell manuscript (Table S1, page 33 of supplemental material), we retrieved the metastasis site annotation from Table 8 of Bos, P., et al. "Genes that mediate breast cancer metastasis to the brain" Nature. 2009, 459: 1005-9. The metastasis site annotation was reported for 560 of the 615 samples. The median duration of follow-up was 7.667 years (range, 0 to 14.25) for the 268 patients without metastasis and 1.917 years (range, 0 to 9.583) for the 292 patients with metastasis. The median follow-up for all 560 patients was 4 years (range, 0 to 14.25). Those 55 patients lacking of time to metastasis annotation were not included in any ulterior time to metastasis analysis.

To examine the prognostic value of RARRES3 in different subsets of breast cancers, we divided the breast cancer samples on the basis of their ER status. For ER status, we used the intensity of ESR1 on the Affymetrix chip, as the pathological status was not available (for GSE12276). The distribution of ESR1 gene showed strong bimodality. We defined the ER+ and ER- tumors based on this bimodality. We defined the ER+ and ER−tumors based on this bimodality (ER+ BC n=349).

Missing Values Report

Five hundred and sixty patients are represented in the cohort. Of which, the variables “ER, “HER2mod”, “tumor size” have 15(2%), 20 (3%), and 63 (11%) missing values, respectively.

RARRES3 three-dimensional structural analysis

The homology model of RARRES3 was constructed using the I-TASSER (Roy et al., 2010). This model was then minimized using the charm22 GBSW implicit solvent model (Chen et al., 2006) with distance restraints on the heavy side chain atoms for the catalytic residues to ensure that their orientation from the structure of H-REV107 (PDB ID 2KYT) was conserved (Ren et al, 2010). Finally, the structure was minimized without restraints using the KOBA knowledge-based potential (Chopra et al., 2010). The quality of the model was verified using MolProbity (Chen et al., 2010). Structures were visualized with Pymol, and the sequence alignment was generated with Alscript (Barton, 1993).

Supplementary References

Barton, G.J., 1993. ALSCRIPT: a tool to format multiple sequence alignments. Protein Eng 6, 37–40.

Chen, J., Im, W., Brooks, C.L.r., 2006. Balancing solvation and intramolecular interactions: toward a consistent generalized Born force field. J Am Chem Soc 128, 3728–3736.

Chen, V.B., Arendall, W.B.r., Headd, J.J., Keedy, D.A., Immormino, R.M., Kapral, G.J., Murray, L.W., Richardson, J.S., Richardson, D.C., 2010. MolProbity: all-atom structure validation for macromolecular crystallography. Acta Crystallogr D Biol Crystallogr 66, 12–21.

Chopra, G., Kalisman, N., Levitt, M., 2010. Consistent refinement of submitted models at CASP using a knowledge-based potential. Proteins 78, 2668–2678.

Ren, X., Lin, J., Jin, C., Xia, B., 2010. Solution structure of the N-terminal catalytic domain of human H-REV107--a novel circular permutated NlpC/P60 domain. FEBS Lett 584, 4222–4226.

Roy, A., Kucukural, A., Zhang, Y., 2010. I-TASSER: a unified platform for automated protein structure and function prediction. Nat Protoc 5, 725–738.
